# Visual and anatomical outcome of macular hole surgery at a tertiary healthcare facility

**DOI:** 10.12669/pjms.335.13089

**Published:** 2017

**Authors:** Komalta Kumari, Muhammad Ali Tahir, Alyscia Cheema

**Affiliations:** 1 Dr. Komalta Kumari, FCPS (Ophth), MRCSEd(Edin), Department of Ophthalmology, Jinnah Post Graduate Medical Centre, Karachi, Pakistan; 2 Dr. Muhammad Ali Tahir, FCPS (Ophth), FCPS (VR), Department of Ophthalmology, Jinnah Post Graduate Medical Centre, Karachi, Pakistan; 3 Dr. Alyscia Cheema, FCPS (Ophth) FRCS (Edin), Department of Ophthalmology, Jinnah Post Graduate Medical Centre, Karachi, Pakistan

**Keywords:** Brilliant Blue G Dye, ILM peeling, Macular hole, Vitrectomy

## Abstract

**Objective:**

To assess visual and anatomical outcome of full thickness macular hole (FTMH) surgery with ILM peeling using brilliant blue G dye.

**Methods:**

Thirty patients who had clinically evident macular hole were selected. Pre-operative Optical Coherence Tomography (OCT) was done. In all cases vitrectomy was performed via 23guage 3 ports pars plana (3PPV) vitrectomy system and Brilliant blue G dye, 0.5ml dye was injected over macula which resulted in light blue stain of ILM and peeling was performed around hole in circular motion and after gas fluid exchange gas tamponade with SF6 was done. Final visual and anatomical outcome was measured as postoperative BCVA and postoperative OCT at three months respectively. Descriptive statistics were computed. Paired t-test was applied. P value≤0.05 were considered as significant.

**Results:**

There were 12 male and 18 female patients. The mean age was 57.40±4.76 years. The mean size of macular hole was 452.20±242.33μm. The mean duration of symptoms was 16.73±13.49 weeks. Mean pre operative BCVA was 1.30±0.73 log MAR and post operative was 0.51±0.23 log MAR. Mean increased BCVA was found to be 0.22±0.13 log MAR. Primary closure of hole was achieved in 29(96.7%). Significant mean difference was found in pre operative and post operative BCVA.

**Conclusion:**

Brilliant blue G exhibits sufficient staining qualities and safety profile to peel ILM in the management of full thickness macular hole with significant visual and anatomical improvement.

## INTRODUCTION

A macular hole is a defect at the fovea with interruption of all retinal layers (except stage 1A and 1B) from the internal limiting membrane (ILM) up to the Retinal Pigment Epithelium (RPE).^1^ Macular hole is an important cause of central visual loss and the overall prevalence is approximately 3.3 per 1000 and strong female predominance.^2^,^3^

Macular hole can be associated with trauma or myopia but most common cause is idiopathic. Idiopathic macular hole are commonly seen in women in the seventh decade of life without any apparent predisposing conditions.^2^-^4^ Classic macular hole surgery consists of vitrectomy, posterior vitreous cortex separation and intraocular gas tamponade. During the past decade, focus has been on ILM peeling as adjuvant therapy for increasing closure rates.^5^

Kelly and Wendel introduced a surgical procedure to close macular holes. They achieved an anatomical closure rate of 73% and visual improvement of two or more lines.^6^ During the last decade closure rates have improved significantly due to improved surgical techniques.^7^ According to literature, pars plana vitrectomy is a recommended treatment for stage 2,3 and 4 full thickness macular hole(FTMH). Primary closure rate of FTMH is achieved better with ILM peeling. ILM can be stained with Brilliant blue G dye for better visualization and complete removal of traction around the hole.^8^ Gupta B and colleagues had the anatomical success rate of 86% and variable visual success rate.^9^ Brooks reported 100% closure in holes of less than 6 months duration with ILM peeling.^10^ The purpose of this study was to see the anatomical and visual results in our circumstances.

## METHODS

Total 30 patients with idiopathic FTMH were selected from retina clinic in ophthalmology department of Jinnah Postgraduate Medical Center, Karachi, Pakistan from January 2015 to June 2016. After detailed history and ophthalmic examination (IOP measurement, Watzke Allen test, Amsler grid, fundoscopy using +90 Diopter lens and indirect ophthalmoscopy) patients who had clinically evident macular hole and after exclusion of other peripheral retinal tears were selected for 23 guage 3PPV. Pre-operative Swept Source OCT was done for further staging of Hole, measurement of hole size, to differentiate from simulating lesions, to identify lamellar holes, Vitreo macular taction, presence of Epiretinal membrane(ERM) and subretinal fluid.

Study was done after approval from institutional ethical review committee. Informed consent was obtained prior to procedure. Vitals were checked. Intravenous line was maintained. Pupils were dilated using Tropicamide 1% and phenylephrine 10%, peribulbar anesthesia with lidocaine 2% with adrenaline was administered. Povidone iodine 5% was used to clean surgical site and drape and opsite applied. Three ports made at 4mm and 3.5mm from limbus in phakics and pseudophakics respectively. Using 23guage 3PPV system, core vitrectomy was performed. After that triamcinolone was used to stain vitreous and posterior vitreous detachment induced and vitrectomy completed. Brilliant blue G (DORC, international), 0.5ml, dye was injected over macula under BSS which resulted in light blue stain of ILM and peeling was performed around hole in circular motion with the help of ILM peeling forceps via pinch and squeeze method. Then Gas Fluid exchange was performed, intraocular gas tamponade with SF6 was done. Postoperatively no specific head down posturing was advised for five days and topical combination of antibiotic and steroid was given. IOP was monitored. Postoperative OCT was done to assess anatomical closure of hole at one month and 3 months interval. Final visual outcome was measured as postoperative BCVA at three months.

### Statistical Analysis

Data was analyzed by using statistical package for social sciences (SPSS) version 21. Mean and standard deviation were computed for quantitative variable and frequency and percentage were calculated for qualitative variables. Paired t-test was applied to compare pre and post operative means of best corrected visual acuity (BCVA). P value≤0.05 were considered as significant.

**Fig.1:**
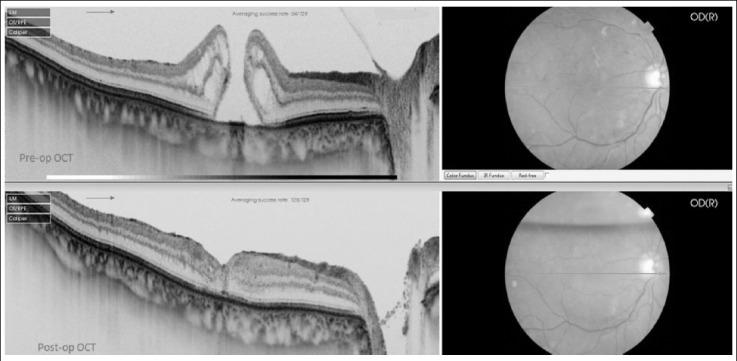
Pre-op OCT showing stage 4 FTMH, Post-op OCT showing closure of FTMH at 2 weeks.

## RESULTS

The results showed that there were 12 male and 18 female patients. The mean age of patients was 57.40±4.76 years. Size of macular hole was found 452.20±242.33 μm. The mean duration of symptoms was 16.73±13.49 weeks. Most of the patients 20(66.7%) were phakic eyes while most of the patients 13(43.3%) have stage two of macular hole. Mean pre operative BCVA was 1.30±0.73logMAR and post operative was 0.51±0.23logMAR. Mean increased BCVA was found to be 0.22±0.13logMAR. Primary closure of hole was achieved in 29(96.7%). Complication after Macular Surgery was found in 19(63.3%) patients. Most common complication was cataract as presented in Table-I. Detailed characteristics of patients are shown in Table-I.

**Table-I:** Characteristics of patients.

	*n (%)*
Age(years)^0^	57.40±4.76
Size of macular hole(μm)^0^	452.20±242.33
Duration of symptoms(weeks)^0^	16.73±13.49
Preoperative BCVA^0^	1.30±0.73
Post operative BCVA^0^	0.51±0.23
Increased BVCA^0^	0.22±0.13
*Gender*	
Male	12(40)
Female	18(60)
*Lens Status*	
Phakic	20(66.7)
Pseudophakic	10(33.3)
*Stages of macular hole*	
Stage 2	13(43.3)
Stage 3	11(36.7)
Stage 4	6(20)
*Primary closure of hole achieved*	
Yes	29(96.7)
No	1(3.3)
*Complication after Macular Surgery*	
Cataract	11(36.7%)
Raised IOP	3(10%)
Punctuate hemorrhages	5(16.7%)
Cataract & Punctuate hemorrhages	2(6.7%)
Cataract and reopening of hole	1(3.3%)
None	11(36.7)
ERM	2(6.7%)

0 Mean±SD

Significant mean difference was found in pre operative and post operative BCVA (P-value < 0.01). Significant mean difference in pre operative BCVA and post operative BCVA was also observed among patients with stage 2(P-value < 0.01) and stage 3(P value < 0.01). Detailed results of comparison of mean differences in pre operative BCVA and post operative BCVA among various demographic and clinical characteristics are presented in Table-II.

**Table-II:** Visual increase with respect to various demographic and clinical characteristics.

	*N*	*Pre Operative BCVA*	*Post Operative BCVA*	*P-Value*	
Overall	30	0.737±0.294	0.510±0.387	0.000	P<0.01
***Gender***					
Male	12	0.825±0.282	0.614±0.398	0.000	P<0.01
Female	18	0.678±0.295	0.441±0.375	0.000	P<0.01
***Size of macular hole***					
≤400µm	13	0.469±0.143	0.176±0.116	0.000	P<0.01
>400µm	17	0.942±0.197	0.765±0.321	0.000	P<0.01
***Duration of symptoms***					
≤ 16 weeks	17	0.558±0.212	0.258±0.197	0.000	P<0.01
> 16 weeks	13	0.973±0.214	0.839±0.324	0.002	P<0.01
***Lens Status***					
Phakic	20	0.660±0.281	0.423±0.347	0.000	P<0.01
Pseudophakic	10	0.891±0.270	0.684±0.423	0.003	P<0.01
***Stages of macular hole***					
Stage 2	13	0.4692±0.143	0.1769±0.116	0.000	P<0.01
Stage 3	11	0.912±0.218	0.724±0.325	0.001	P<0.01
Stage 4	6	0.996±0.157	0.840±0.328	0.097	P>0.01
***Complication after Macular Surgery***					
Yes	19	0.832±0.287	0.642±0.398	0.000	P<0.01
No	11	0.572±0.237	0.281±0.244	0.000	P<0.01

Paired t-test applied. P-value<0.05 considered as significant.

## DISCUSSION

The anatomical success rate for this study was 96.7% which is approximately similar to other studies.^2^,^9^,^11^ Out of 30 holes only one hole reopened despite surgery. Some times more than one surgery is required to achieve the anatomical success.^12^ New surgical adjuvant and techniques decreases surgical time and increased success rate. One of the best examples is peeling of ILM as a treatment for idiopathic macular holes. Increase anatomical success and prevent reopening of the hole by decreasing ERM development was reported for ILM peeling.^13^ Studies have also suggested increased anatomical success but not functional success for ILM peeling.^14^ ILM peeling is recommended especially in patients with a hole wider than 400 µm.^15^

In this study, we performed macular hole surgery on 30 patients, all underwent ILM peeling assisted with BBG staining. Out of the 30 patients, 26 (86.66%) had a recordable visual increase which is same as reported by Khaqan HA et al.^16^ Another study reported a mean increase in BCVA of 0.23±0.01 log MAR and visual improvement in 82% patients.^17^ The BCVA remained

unchanged in four patients in our study. The results of this study are promising for a better visual outcome and we believe ILM peeling leads to good visual outcome in patients with macular hole. Report of Kelly and Wendal in 1991 changed the concept about macular hole as an untreatable blinding disease.^18^ Studies reported significant association of ILM peel with anatomical and functional improvement.^19^

In this study, mean visual increase was found 0.22±0.13log MAR. Significant mean difference between Pre operative BCVA and post operative BCVA was also found for patients with stage two and stage three. A previous study reported mean visual increase of 0.27±0.08, 0.23±0.04 and 0.14±0.07 in stage 2, 3 and 4, respectively.^20^

**Fig.2:**
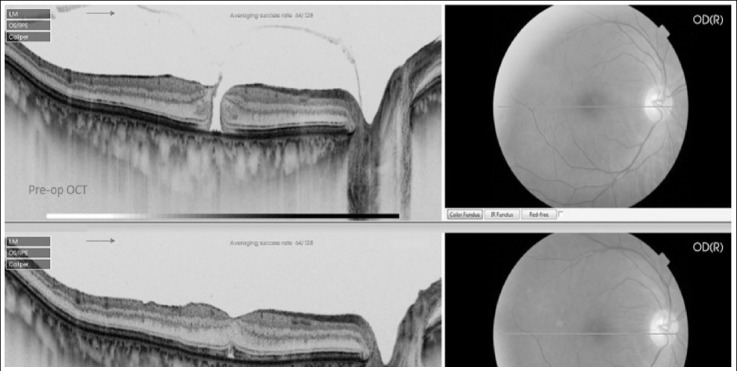
Pre-op OCT showing stage 3 FTMH, Post-op OCT showing closure of FTMH at 4 weeks.

Shukla et al., compared trypan blue(TB), BBG and indocyanine green(ICG) dyes in their ease in ILM peeling which evaluated the better visual outcome after PPV and ILM peeling assisted with BBG staining in cases of macular hole.^17^ Shimada et al have conducted a prospective, interventional study to evaluate the usefulness of BBG. The ERM recurrence rate was reduced to 0% in eyes with double ERM and ILM peeling compared with 16.3%, and the reoperation rate was 5.8% that underwent single ERM peeling. The ERM peeling methods differed in the rate and extent of residual ILM, and the lowest rate (39%) was achieved with BBG staining (P<.0001).^21^

A study comparing BBG, TB, and ICG showed that, in eyes with macular holes stages 3-4, there was no statistically significant difference in anatomical closure rates. At six months postoperatively, there were significantly more eyes in the combined BBG and TB group that had visual improvement in comparison to the ICG group. Also, visual acuity deterioration was significantly more common in the ICG group. That study reported that the participating surgeons described better ILM staining with BBG compared to TB, as well as easier ILM removal. Those results are also in compliance with the results of the current study.^22^ In another study, it was observed that the mean preoperative best-corrected visual acuity (BCVA) was 0.7 log MAR units (mean ± SD 0.66 ± 0.27). After 3 months, the mean BCVA increased not significantly to 0.4 (0.54 ± 0.30), but a significant improvement to 0.2 log MAR units (0.28 ± 0.23) could be detected after 6 months compared to baseline (p < 0.01).^23^

The prevalence of idiopathic macular hole in general population is 7.8/100,000 and it shows a strong female predominance. During the study period, only 30 patients could be recruited because of the lower prevalence of disease. The small sample is a limitation of this study. No iatrogenic retinal tear and retinal detachment was observed in our patients postoperatively.

### Strength of this study

Though lot of work has already been done on the subject of Macular hole in different context but our work specifically add and support the following facts:

1. In our research work we have used SF6 which is a short acting gas tamponade and observed hole closure even in medium to large size full thickness macular hole(FTMH) without specific head posturing so this gives confidence to a surgeon to use short acting gas like SF6 in medium to large FTMH. SF6 gets absorbed in two weeks and by that period of time hole also gets closed without increase in chance of reopening of hole after the absorption of the gas. However in most of the previous work C2F6 or C3F8 long acting gas tamponades have been used with head down posturing for medium to large macular hole to get successful hole closure.
2. In previous research work ICG dye was used to stain ILM for peeling but retinotoxicity was reported by some authors..
3. Our study supports and strengthens those studies that used Brilliant Blue(BBG) dye to stain ILM and observed excellent staining comparable to ICG without observing retinotoxicity. Also we used BBG dye under balance salt solution that reduces the time of surgery as compared to ICG which needs to be used under air which require gas fluid exchange, increasing the time of surgery

## CONCLUSION

In view on the results of our study, it can be concluded that brilliant blue G is an effective adjunctive tool for ILM peeling. It has sufficient staining qualities and safety profile leading to a significant functional and anatomical improvement after successful macular hole surgery.

### Authors’ Contribution

**KK** Literature search, acquisition of data, drafting the article and final approval of manuscript.

**MAT** Designed the study, acquisition of data, performed surgeries.

**AC A**nalysis, critical review and final approval of manuscript and supervised the study.
